# 

**Published:** 1884-07

**Authors:** 


### CONTENTS:

Art. I.—Some of the Drugs Used in Dentistry.—By Charles W. Glassington, M. R. C. L., S. D. S. .....97
Art. II—The Higher Education. By J. D. Moody.....108
Art. III—Irregularity—A Practical Case. By Charles P. Weinrich, D. D. S. .....117
Art. IV.—Surgical Delusion. By John B. Roberts, M. D. .....122
Art. V.—Neuralgia of Dental Origin.—Its Discrimination and Treatment, By J. Morley Dennis.....127
Art. VI.—How to Secure Good Dental Organs, Etc. By H. E. Dennett. D.D.S. .....132
Art. VII.—Chromic Acid in Affections of the Tongue.....134
American Dental Association.....135

### EDITORIAL, ETC.

Answer to An Unprovoked Attack.....136

Information Wanted.....138

The New Building of the Dental Department of the University of Maryland.....139

### MONTHLY SUMMARY.

A Dental Bill.....140
Textile Metallic Filling.....141
Acid Dyspepsia.....142
Germs and Germicides.....143

